# Psychotraumatology: rich in international and cross-cultural collaboration

**DOI:** 10.3402/ejpt.v4i0.21259

**Published:** 2013-06-06

**Authors:** Miranda Olff

**Affiliations:** Department of Psychiatry, Academic Medical Center, University of Amsterdam & Arq Psychotrauma Expert Group, Diemen, The Netherlands

**Keywords:** Trauma, PTSD, European psychotraumatology, international, cross-cultural collaboration, TENTS, EJPT

## Abstract

Although European countries differ widely in their approach to psychotrauma and its consequences, with 20 years of European psychotraumatology in the form of the European Society for Traumatic Studies (ESTSS) there is now increasing dissemination of evidence-based care reaching more and more professionals and scholars in Europe. The society has moved forward to become a European umbrella organization serving many different European countries and cultures. This article describes the process of the restructuring and how ESTSS has grown from collaboration with other traumatic stress partners inside and outside Europe. The knowledge generated by The European Network for Traumatic Stress (TENTS) project has helped this process as well as the creation of an international open-access journal, the *European Journal of Psychotraumatology* (*EJPT*), for optimal dissemination of knowledge.

Clinicians and researchers in Europe in the field of trauma and PTSD have become increasingly aware of the need for consensus regarding evidence-based effective interventions for psychotrauma-related disorders. Although European countries differ widely in their approach to psychotrauma and its consequences (Witteveen et al., 2012), with 20 years of European psychotraumatology in the form of the European Society for Traumatic Studies (ESTSS) there is now increasing dissemination of evidence-based care reaching more and more professionals and scholars in Europe. Congratulations on the 20-year anniversary of ESTSS (Lueger-Schuester, 2013).

My first memory of ESTSS stems from the ESTSS conference in Edinburgh in 2001. There was a great scientific program, very helpful for a newcomer in the field, but it is interesting to note that sometimes the social and cultural events so typical for ESTSS meetings seem to be remembered best. In this case, I have a vivid memory of serious scientists dancing in Scottish kilts. In all, it has been a pleasure to have been part of this warm and inspiring society which has also very much shaped my career. Below I will describe what I have felt as important tasks for ESTSS and how I tried to move the society forward during my years on the ESTSS board. This comes down to implementing the restructuring of the society, coordinating The European Network for Traumatic Stress (TENTS), and starting an open-access scientific journal: the *European Journal of Psychotraumatology* (*EJPT*).

I joined the board of the ESTSS in 2005, being introduced by Berthold Gersons while working at the Department of Psychiatry, Academic Medical Center, with a small team focusing on trauma and PTSD. I started as treasurer, from 2007 was the secretary, and in June 2009 I became president of ESTSS, the first female president! A nice past president's picture taken at the ESTSS conference in Stockholm shows my all male predecessors (see supplementary material). But this trend has now been changed. After me, Brigitte Lueger Schuester helped to make up for the gender imbalance, now slightly better reflecting the population we are serving (Olff, Langeland, Draijer, & Gersons, 2007).

## Restructuring ESTSS into an umbrella organization

With the start of my presidency in 2009—after long strategic discussions on the restructuring which Berthold Gersons and Jon Bisson before me had graciously started (see also Gersons, 2013, and Bisson, 2013)—the ESTSS officially became the European umbrella organization: an organization consisting of local or regional European STSS member and affiliated societies (Table 1).

**Table 1:** ESTSS membership structure from 2009 (see also www.estss.org/about/membership-types)

With the umbrella structure ESTSS membership consists of: All members from member societies (local STSSs) Member societies are local, regional- or language-based traumatic stress societies who share the aims of the ESTSS. There is a formal agreement that the entire membership of the Member society will become members of ESTSS Individual members from affiliated societies Affiliated societies are societies that cannot join as member society or partially share the aims of ESTSS. There are no fees for the society, but it allows its individual members to join ESTSS at a reduced rate Regular individual members

This led to an exciting and challenging organizational adventure of trying to cover Europe and in particular to include more Eastern and Southern European countries. It was a very rewarding job as the ESTSS expertise was warm heartedly received by new organizations that we have been helping to start up (see slides in the supplementary material). It was also an interesting experience to learn to appreciate different cultures, societal and scientific, from serious training and teaching to (a sip of) vodka with the director in the basement of a beautiful classical music hall.

Europe is rich in its diversity, and my “Dutch” evidence-based, straightforward approach sometimes needed a bit more time and tact, but in the end I believe we came a step further towards European psychotraumatology. New member societies to ESTSS came from France (AFORCUMP-SFP), the German-speaking countries (DeGPT), Switzerland (SSPT), Italy (SISST), Denmark (The Danish Society of Psychotraumatology), Portugal (Centro de Trauma), and Croatia (Croatian Society for Traumatic Stress). Newly affiliated to ESTSS were organizations from Norway (NKVTS and RVTS), Spain (EMDR Spain), Belgium (BIPE), Ukraine (ECP), Lithuania (Psychological Association Lithuania), Sweden (EMDR Sweden) and Austria (Austrian Psychological Association). I also felt it was important to have strong links with other overarching European societies and that members would benefit from discounts from each other's meetings. Therefore, we also affiliated with EMDR Europe, ESTD, ESSPD, TENTS, EFPA standing committee on trauma and disasters, and the International Red Cross. Newly affiliated Non-European societies are the Japanese Society for Traumatic Stress Studies, and The World Society of Victimology (WSV). For the full and current listing, see www.estss.org

## New website

In parallel to the restructuring, we have created a completely new website—with a new logo and fresher look—containing up-to-date information on the new structure, the participating societies, the conferences, literature, and other news and information on member benefits such as access to the *ISTSS Journal of Traumatic Stress* and the free publication in the *EJPT*.

The <ESTSS history page> “About” section has been very helpful refreshing my memory for this article!

## Collaboration with other societies

It has been very rewarding to not only connect to or help establish member and affiliated societies in Europe, but also to affiliate with our colleagues in Australasia and Japan. Important also are the collaborations that come from affiliations with other European societies such as ESTD, ESTPD, and EMDR societies (e.g., joint days on the biennial conference), which help to unite the field and improve collaboration.

There is constructive collaboration with ISTSS whose board meetings often take place during the ESTSS conference and whose members help to provide popular keynotes and workshops. There has always been a close connection between ESTSS and ISTSS, sometimes perceived as “productive rivalry” (Gersons, 2013). Theoretically, ISTSS should be the mother society of all STSSs worldwide, but this has not been easy to realize. In reality, ISTSS has not been fully fulfilling its international role in the sense of equally serving members all over the world and has a predominantly North-American membership (see also Schnyder, 2013). However, recognizing that traumatic stress is a global issue, ISTSS with Ueli Schnyder as the lead, decided to seek to have a stronger global impact on trauma-related issues. My background in ESTSS is hopefully helpful for my task as the current vice-president of ISTSS in increasing global collaboration and helping ISTSS become a truly international society.

## Global collaboration

Another way to increase international partnership is the Global Collaboration initiative that I am currently chairing. It is fantastic to experience how ISTSS, ESTSS and other ISTSS affiliate societies agreed to work alongside each other on an equal basis, identify objectives, facilitate development, and coordinate activities of global importance.

In 2012, the Global Collaboration will focus on the impact of *childhood abuse and neglect* in adults. Very briefly, the aim is to synthesize a core guideline for prevention and treatment that can be customized for specific cultural contexts worldwide and to disseminate these guidelines using a mobile app that will allow for worldwide distribution and cultural customization (for more information, see Schnyder & Olff, in press; Koenen, 2013). The first grant application has now been written.

## The European Network for Traumatic Stress

The restructuring fitted very well with the TENTS project where we have been aiming to improve evidence-based care across Europe with a large consortium of European researchers and clinicians, a project started and supported by ESTSS board members. TENTS consisted of nine associated partners (i.e. countries) and six collaborating partners throughout Europe, see also www.estss.org/tents).

The TENTS project initially focused on interventions in the aftermath of disaster and implementation of these interventions, later broadened to all trauma types. It was recognized that many countries and regions throughout Europe have limited expertise available and lack the capacity to effectively respond to psychosocial needs in the aftermath of disasters. The European Union (EU) acknowledged these issues and demonstrated its support by co-funding the TENTS project. The general purpose of TENTS was to build Europe-wide networks of expertise on the psychosocial management of victims of natural and other disasters, and to help mental health services of provincial and district health authorities develop into more evidence-based and effective services.

TENTS resulted in the development of evidence-informed post-disaster psychosocial management guidelines (Bisson et al., 2010). Is also resulted in a paper describing the availability of post-disaster psychosocial services across Europe and how these compare to the evidence-informed psychosocial care guidelines (Witteveen et al., 2012). It showed that countries in East Europe seemed to have less central coordination of the post-disaster psychosocial response, that several forms of psychological debriefing, for which there is no evidence of efficacy to date and some evidence of potential harm, were still used in several areas particularly in North Europe. East European countries delivered evidence-based interventions for PTSD less frequently, whilst in South- and South-Eastern European countries, anxiolytic medication such as benzodiazepines were prescribed more frequently than in other areas. For more details, see Witteveen et al. (2012).

TENTS-TP (Training and Practice), a second EU-funded program, was broadened to all types of traumatic events and has been focusing on the implementation of knowledge in the form of training and practice across Europe. It is still ongoing with a dedicated group of partners now within the General Certificate of Psychotraumatology (see Bisson, 2013). These projects have helped to integrate European psychotraumatology and to stimulate cross-cultural and international collaboration.

## European Journal of Psychotraumatology

With the dissemination of knowledge of evidence-based practice a high priority, in December 2010, ESTSS launched its own journal: *EJPT* (www.eurojnlofpsychotraumatol.net) with both scientific papers and more clinical practice-oriented contributions. The journal is in line with ESTSS's mission to advance and disseminate scientific knowledge about traumatic stress within Europe and internationally, and it is intended as a forum for the publication of peer-reviewed original research papers as well as to serve as a forum to disseminate evidence-based clinical practice.

I thought it was particularly important that the journal would be open access, reflecting ESTSS's stand on free accessibility of research publications to a wider community via the web. The Dutch research council (NWO) provided a grant to help the journal start up and to make it possible to waive publication fees for the first 3 years. The open-access policy is increasingly embraced, and in the future it will become mandatory to publish open access on research funded by the EU or other public money.

Being editor-in-chief of *EJPT* has been a very exciting endeavor. We have a great team of associate editors (Vittoria Ardino, Chris Brewin, Marylène Cloitre Ruth Lanius, Agnes van Minnen, Rita Rosner, and Stuart Turner), as well a large international editorial board of psychotrauma experts. Now after 2 years, it has already been indexed in major databases, including PubMed, and has an unofficial impact factor of 1.51 (for its development, see also Olff, 2010, 2012; Olff and Bindslev, 2011). Again, this is the result of a large number of professionals from many different countries, both within and outside Europe, who have devoted time and effort help the journal start up.

## ESTSS conferences

My presidency ended in 2011 at the ESTSS conference in Vienna. Here, I handed the presidency over to Brigitte Lueger Schuester, and she will hand over to our Turkish colleague, Vedat Sar, at the conference in Bologna.

ESTSS conferences are attended by between 800 and 1,200 participants from all over Europe as well as from the United States, South-America, Asia, Africa, and Australia. Typically, local groups organize these European conferences, usually with active support from local government. The diversity of culture, food, and music helps to make these conferences very attractive meeting places for trauma professionals to get to know each other as well as to exchange experiences and present their scientific and clinical expertise.

In summary, I hope to have shown that European psychotraumatology has a rich history in international and cross-cultural collaboration and that by uniting forces, bringing together countries and cultures, we are stronger and wiser. I look forward to the next 20 years of ESTSS. And nobody should miss the birthday party at the ESTSS conference in Bologna!
